# ACEI/ARB therapy in COVID-19: the double-edged sword of ACE2 and SARS-CoV-2 viral docking

**DOI:** 10.1186/s13054-020-03195-9

**Published:** 2020-07-31

**Authors:** Chidinma L. Onweni, Yu Shrike Zhang, Thomas Caulfield, Christopher E. Hopkins, De Lisa Fairweather, William D. Freeman

**Affiliations:** 1 Department of Critical Care Medicine, Mayo Clinic, Jacksonville, FL USA; 2 Division of Engineering in Medicine, Brigham and Women’s Hospital, Boston, MA USA; 3 Department of Neurologic Surgery, Mayo Clinic, 4500 San Pablo Rd, Jacksonville, FL 32224 USA; 4 Department of Health Sciences Research, Mayo Clinic, Jacksonville, FL USA; 5 Brigham and Women’s Hospital, Boston, MA USA; 6 InVivo Biosystems, Eugene, OR USA; 7 Department of Cardiovascular Medicine, Mayo Clinic, Jacksonville, FL USA; 8 Department of Immunology, Mayo Clinic, Jacksonville, FL USA

**Keywords:** ACE2, ACEI/ARB therapy, COVID-19

The use of angiotensin-converting enzyme inhibitors (ACEIs) and angiotensin receptor blockers (ARBs) in patients with severe infection from coronavirus disease 2019 (COVID-19) has been the subject of considerable debate. The question is whether these drugs are harmful or helpful in the therapeutic management of the disease.

ACEIs and ARBs act on the renin-angiotensin-aldosterone system (RAAS) by attenuating the hypertensive effects of angiotensin II (Fig. 1) [1–4]. One of the body’s natural angiotensin II attenuators is angiotensin-converting enzyme 2 (ACE2), an extracellular transmembrane enzyme that is responsible for breaking down angiotensin II into the angiotensin-(1-7) heptapeptide. Yet, ACE2 is the main receptor for binding and uptake of severe acute respiratory syndrome coronavirus 2 (SARS-CoV-2) into the cell. Indeed, in vitro data support the concept that respiratory epithelium, which appears to be the main route of SARS-CoV-2 entry into the body, has multiple cell types with high ACE2 expression [1]. Viral binding leads to internalization and enzymatic degradation of ACE2, thereby promoting hypertensive effects by increasing angiotensin II levels [2]. ACEIs and ARBs are therapeutic because they block angiotensin II signaling, but their use is known to induce higher expression of ACE2 at the membrane, which could allow increased viral entry, especially into the lungs, heart, and kidneys [2]. The debate was fueled further by clinical data from Zhang et al. [4], who reported that all-cause mortality for patients with COVID-19 was lower among patients taking ACEIs/ARBs compared with patients not taking those drugs. Those findings prompted a statement from various medical societies advising physicians to continue to follow current guidelines for using these drugs in virus-positive patients hospitalized for COVID-19 [4].

**Fig. 1:**
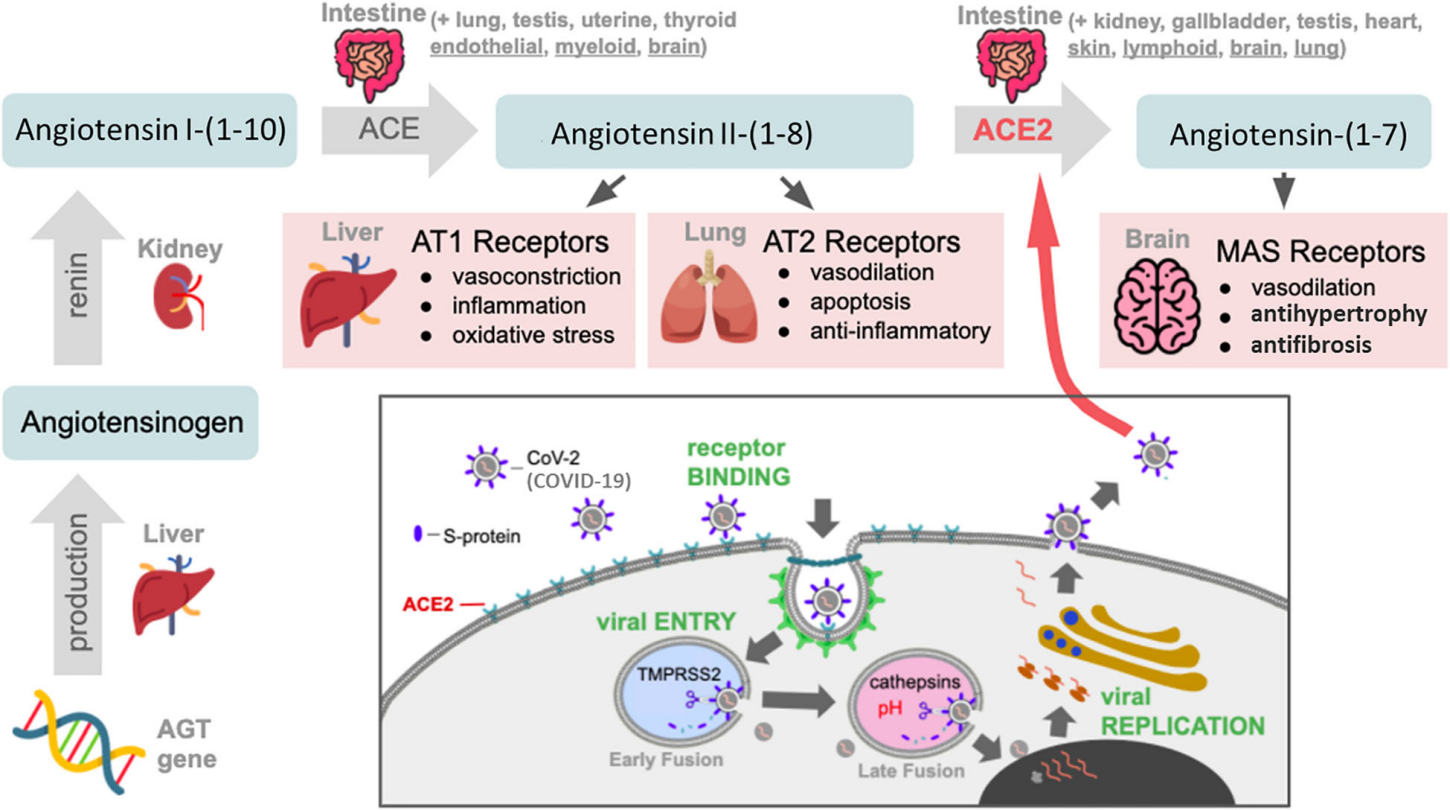
Model for engagement of renin-angiotensin-aldosterone system with severe acute respiratory syndrome coronavirus 2 (SARS-CoV-2). The virus binds angiotensin-converting enzyme (ACE) 2 (ACE2), promoting internalization of the viral receptor. ACE2-dependent production of angiotensin-(1-7) is disrupted, and production of angiotensin II-(1-8) increases. Changes in angiotensin levels alter the target receptor activity in select tissues. Major organs for gene expression are represented with images, and sites for secondary expression are listed in parentheses. Underlining indicates that the data are from only cell-line analysis. AGT indicates angiotensinogen; AT1, angiotensin II type 1 receptor; AT2, angiotensin II type 2 receptor; CoV-2, coronavirus 2; COVID-19, coronavirus disease 2019; MAS, mitochondrial assembly; TMPRSS2, transmembrane serine protease 2. Expression data were sourced from the Human Protein Atlas (https://www.proteinatlas.org); organ icons made by Vitaly Gorbachev, Smashicons, Prettycons, and Freepik from www.flaticon.com

It seems counterintuitive to use ARBs to upregulate ACE2 as a therapy, while SARS-CoV-2 downregulates ACE2 through viral docking and endocytosis of the ACE2–SARS-CoV-2 complex. However, in animal models, ARB-mediated ACE2 upregulation protects the lungs from coronavirus infection presumably by decreasing downstream ACE-produced angiotensin II and increasing the angiotensin-(1-7) heptapeptide, a potent vasodilator. Although a benefit from the drug is suggested, larger clinical studies of patients with COVID-19 are needed to determine whether the harm outweighs the benefits of administering ACEI/ARB therapy. In addition to these modulators of the RAAS, which can be prescribed or potentially repurposed, recombinant ACE2 enzyme may serve as a potential therapy by binding the virus in the blood [5]. Ultimately, the most successful approach will likely involve polytherapy that interferes with viral uptake and replication and mitigates host-factor comorbidities.

## Data Availability

Not applicable.

## Abbreviations

ACE2: Angiotensin-converting enzyme 2
ACEI: Angiotensin-converting enzyme inhibitor
ARB: Angiotensin receptor blocker
COVID-19: Coronavirus disease 2019
RAAS: Renin-angiotensin-aldosterone system
SARS-CoV-2: Severe acute respiratory syndrome coronavirus 2
